# Emulating Natural Disturbances for Declining Late-Successional Species: A Case Study of the Consequences for Cerulean Warblers (*Setophaga cerulea*)

**DOI:** 10.1371/journal.pone.0052107

**Published:** 2013-01-04

**Authors:** Than J. Boves, David A. Buehler, James Sheehan, Petra Bohall Wood, Amanda D. Rodewald, Jeffrey L. Larkin, Patrick D. Keyser, Felicity L. Newell, Gregory A. George, Marja H. Bakermans, Andrea Evans, Tiffany A. Beachy, Molly E. McDermott, Kelly A. Perkins, Matthew White, T. Bently Wigley

**Affiliations:** 1 Department of Forestry, Wildlife, and Fisheries, University of Tennessee, Knoxville, Tennessee, United States of America; 2 West Virginia Cooperative Fish and Wildlife Research Unit, Division of Forestry and Natural Resources, West Virginia University, Morgantown, West Virginia, United States of America; 3 U.S. Geological Survey, West Virginia Cooperative Fish and Wildlife Research Unit, West Virginia University, Morgantown, West Virginia, United States of America; 4 School of Environment and Natural Resources, Ohio State University, Columbus, Ohio, United States of America; 5 Department of Biology, Indiana University of Pennsylvania, Indiana, Pennsylvania, United States of America; 6 National Council for Air and Stream Improvement, Inc., Clemson, South Carolina, United States of America; Utrecht University, The Netherlands

## Abstract

Forest cover in the eastern United States has increased over the past century and while some late-successional species have benefited from this process as expected, others have experienced population declines. These declines may be in part related to contemporary reductions in small-scale forest interior disturbances such as fire, windthrow, and treefalls. To mitigate the negative impacts of disturbance alteration and suppression on some late-successional species, strategies that emulate natural disturbance regimes are often advocated, but large-scale evaluations of these practices are rare. Here, we assessed the consequences of experimental disturbance (using partial timber harvest) on a severely declining late-successional species, the cerulean warbler (*Setophaga cerulea*), across the core of its breeding range in the Appalachian Mountains. We measured numerical (density), physiological (body condition), and demographic (age structure and reproduction) responses to three levels of disturbance and explored the potential impacts of disturbance on source-sink dynamics. Breeding densities of warblers increased one to four years after all canopy disturbances (vs. controls) and males occupying territories on treatment plots were in better condition than those on control plots. However, these beneficial effects of disturbance did not correspond to improvements in reproduction; nest success was lower on all treatment plots than on control plots in the southern region and marginally lower on light disturbance plots in the northern region. Our data suggest that only habitats in the southern region acted as sources, and interior disturbances in this region have the potential to create ecological traps at a local scale, but sources when viewed at broader scales. Thus, cerulean warblers would likely benefit from management that strikes a landscape-level balance between emulating natural disturbances in order to attract individuals into areas where current structure is inappropriate, and limiting anthropogenic disturbance in forests that already possess appropriate structural attributes in order to maintain maximum productivity.

## Introduction

Ecologists have long appreciated the fundamental role of disturbance in maintaining biodiversity in many ecosystems (e.g., intermediate disturbance hypothesis [1]). This understanding has led to the development of management practices that seek to emulate natural disturbance regimes (hereafter, ENDR), particularly in systems where disturbances have been suppressed or altered, in order to restore biodiversity and improve habitat conditions for vulnerable species [2]. ENDR strategies have been relatively well-established as a method of improving conditions for many declining early successional species [3], [4], however, it is relatively unknown how declining late-successional species may respond to such practices.

Although severe disturbances (e.g., intense fires, volcanic eruptions) within mature forests are known to return entire systems to early successional stages at large scales, less intense disturbances such as wind-throw, tree senescence, and low-intensity fires, have the ability to create more subtle micro-conditions within forests that some late-successional forest species may respond to favorably. One region where interior forest disturbance regimes have been suppressed or altered is the eastern United States. Prior to European colonization, old-growth forests in the eastern U.S. were regularly disturbed by natural events such as windthrow, tree senescence, and fire [5]–[7]. However, since the early 1900s when forests in this region were almost completely cleared for timber and subsequent agricultural opportunities [8], much of the region has regenerated as second-growth forest and interior disturbances are now rare. Fire has become virtually non-existent because of suppression [7], and because <1% of forests are currently in old-growth condition [9], disturbances caused by treefalls (via senescence and wind) occur less frequently and have less impact [10]. Reduction of fire and other natural disturbances has been linked to a number of negative vegetative responses in eastern forests: declines in disturbance-adapted tree species such as white oak (*Quercus alba*) [11], reduction in canopy heterogeneity [12], proliferation of invasive species [13], and a reduction in tree diversity [14]. Concurrently, a number of forest-dependent animal species have undergone steep population declines during this era. These include vulnerable species such as the Indiana bat (*Myotis sodalis*), West Virginia northern flying squirrel (*Glaucomys sabrinus fuscus*), and cerulean warbler (*Setophaga cerulea*) [15]–[17]. Population declines of these species are likely multi-faceted (particularly for migratory species), but some vulnerable late-successional species may require the specific conditions that small-scale disturbances create and may thus be adversely affected by a lack of perturbations in contemporary second-growth forests [17]–[21]. Hence ENDR, via timber harvesting or prescribed fire, has been suggested as a strategy to restore natural patterns to forest environments that were historically shaped by periodic disruptions and to potentially restore habitat conditions required by these species [6], [19], [22].

Birds are an ideal group to use when evaluating how forest succession and the reduction of natural disturbances during the last century has affected wildlife in the eastern U.S., in part because of long-term monitoring programs such as the Breeding Bird Survey (BBS) [23]. Based on BBS data, the regrowth of eastern forests over the past century has been, expectedly, correlated with increasing populations of some avian forest species, such as northern parula (*S. americana*) and blackburnian warblers (*S. fusca*). However, the successional process has also been, seemingly paradoxically, negatively related to population trends of other species that would seem to benefit from what appears to be an increase in breeding habitat, such as the eastern wood-pewee (*Contopus virens*) and Canada warbler (*Cardellina canadensis*) [24]. Perhaps the most notable declining avian species of eastern forests is the cerulean warbler. The cerulean warbler is a Neotropical-Nearctic migratory species that breeds solely in the canopies of deciduous forests in eastern North America and has long been considered to be a prototypical late-successional species [25], [26]. However, despite recent increases in their putative breeding habitat, cerulean warblers are one of the fastest declining passerines in North America; populations declined 3.2%/yr from 1966 to 2003 and the trend has recently worsened to a decline of 4.6%/yr [24]. They are currently listed as a species of conservation concern by the US government [16] and are considered ‘vulnerable to extinction’ by BirdLife International [27]. Contrary to the long-standing paradigm that their preferred habitat is closed-canopy forest, recent evidence suggests that the cerulean warbler's decline may actually be related to a lack of small-scale, interior forest disturbances in their eastern U.S. breeding grounds [21], [28], [29], particularly in the Appalachians, where an estimated 70% of their remaining population breeds [30]. Consequently, ENDR has been suggested as a method of mitigating degraded forest conditions and restoring habitat for cerulean warblers [21], [31]. However the effectiveness of this strategy, as well as the ideal scale and intensity of the disturbances to be emulated, is not known.

Many studies have documented numerical responses of populations (i.e., abundance or density) to anthropogenic disturbance via forest management [e.g.], [ 32,33]. However, our understanding of the mechanisms responsible for numerical responses to environmental perturbations is much more limited. These mechanisms may begin with individual changes in habitat selection, physiology, breeding behavior, and dispersal [e.g.], [ 34,35] and then may be scaled up to population changes in reproductive rates, annual survival rates, and age structure [e.g.], [ 36], [37,38]. Evaluating more than numerical responses is essential because simple use of, or even preference for, a habitat does not necessarily indicate the quality of that habitat [39], [40]. Mismatches between habitat selection and individual fitness have been identified in several taxa, particularly those inhabiting human-modified habitats where ecological processes have been altered recently and rapidly [e.g.], [ 41], [42,43]. Thus, before considering ENDR to be an appropriate strategy for restoring conditions for declining forest species, detailed studies of individual and population-level responses to disturbance are needed to ensure that our actions do not create such a situation.

In this study, we investigated the consequences of emulating natural disturbances for a late-successional avian species, the cerulean warbler. To do so, we experimentally disturbed mature forest stands at various intensities, spanning the range of local disruptions that could occur naturally in mature forests, across the core of the warbler's breeding range in the Appalachian Mountains. We then assessed short-term responses (up to four years) to these manipulations in terms of breeding density, body condition, age structure, and reproductive output. In addition, we explored regional variation in these responses and the potential impacts of emulating disturbance on the source-sink dynamics of cerulean warblers in the Appalachian region using a deterministic population model. Finally, we discuss the implications of our results for cerulean warbler conservation and management.

## Methods

### Study sites

**Figure 1:**
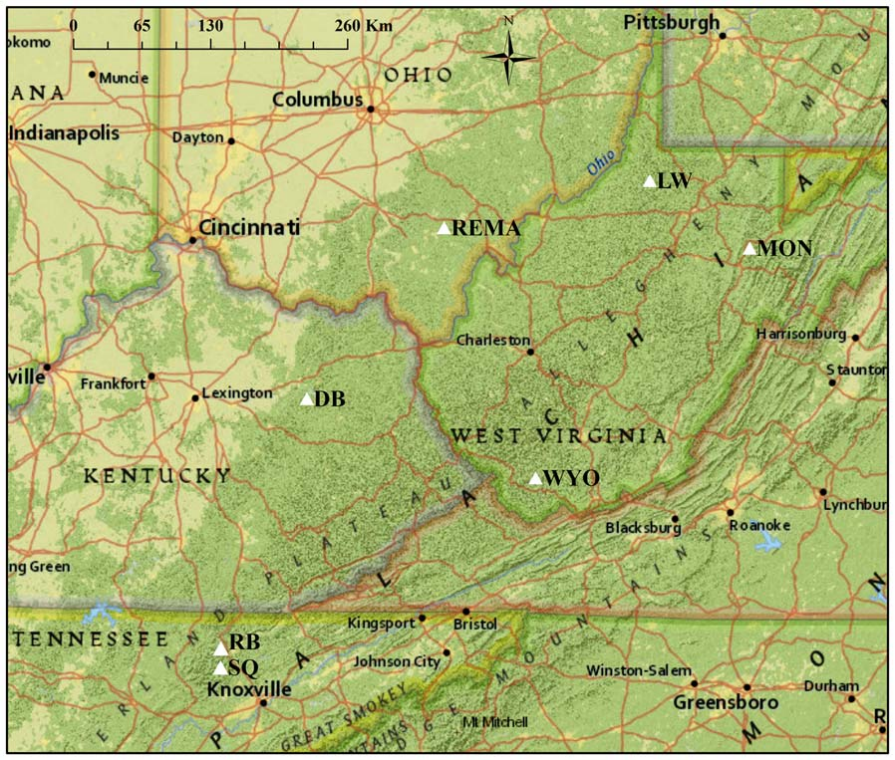
Map displaying locations of seven study sites in the Appalachian Mountains. All sites (white triangles) are located within the core of the cerulean warbler breeding range.

We conducted this study at seven sites in the Appalachian Mountains (Figure 1), all within the Central Hardwoods' mixed-mesophytic forest region [44], which also corresponds to the core of the cerulean warbler breeding range. These sites were: Royal Blue Wildlife Management Area, TN (RB), Sundquist Forest, TN (SQ), Raccoon Ecological Management Area, OH (REMA), Daniel Boone National Forest, KY (DB), Lewis Wetzel Wildlife Management Area, WV (LW), Wyoming County, WV (WYO), and Monongahela National Forest, WV (MON). The two most southern sites (RB and SQ) were both located in the Cumberland Mountains, an ecophysiographically distinct section of the Appalachian chain [45], [46] that has previously been identified as a critical breeding locale for the species [47], [48]. Thus, we refer to these two sites hereafter as the “southern region” and the other five study sites as the “northern region.” Because cerulean warblers often require large tracts of contiguous forest [26], we selected sites embedded within a matrix of mature forest; mean percent forest cover within 10 km of the site center was 83.2±2.8 [SE]% (range = 74–95%, 2001 NLCD). Plant composition differed slightly among sites, but common overstory tree species included tulip poplar (*Liriodendron tulipifera*); sugar maple (*Acer saccharum*); northern red, white, and chestnut oak (*Quercus rubra, Q. alba, and Q. prinus*); and various hickory spp. (*Carya* spp.).

### Disturbance treatments

**Figure 2:**
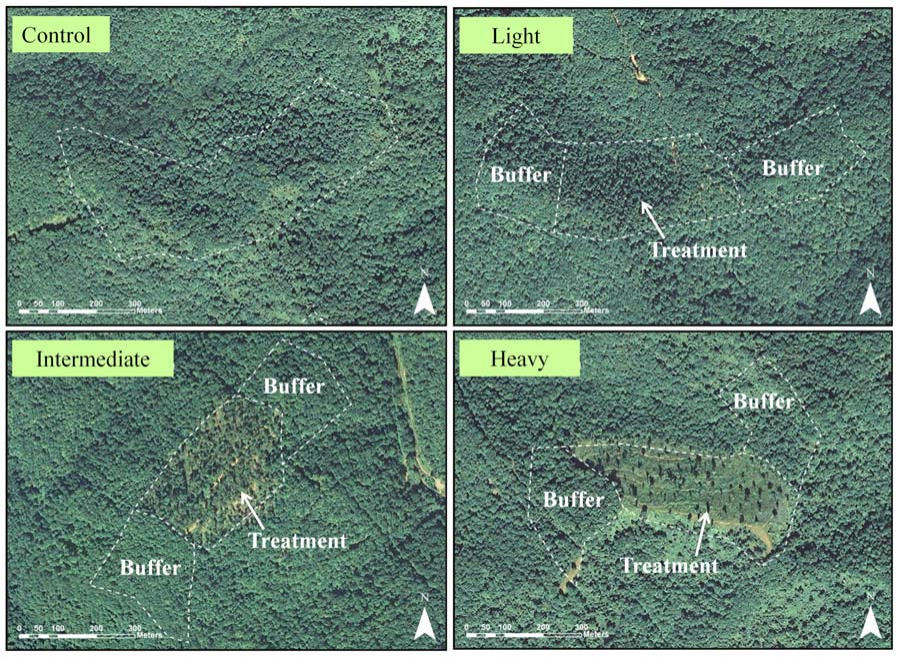
Aerial photos from a study site (LW) depicting treatment plot design and intensity of disturbances. Each field site consisted of three 10-ha treatment plots of various disturbance intensity (created via partial timber harvest) and one 20-ha control plot (undisturbed). Ten ha of undisturbed forest outside the borders of each treatment plot (buffers) allowed for examination of edge effects of the disturbances.

We randomly assigned treatments to four plots at each field site: light, intermediate, and heavy canopy disturbance, as well as an undisturbed control plot. Disturbance plots were 10 ha and control plots were 20 ha in size (Figure 2). We used larger undisturbed control plots because territory density was lower and nests more difficult to locate in these habitats. Each plot was located >200 m from all other plots to maintain independence. At the periphery of each disturbance treatment were two 5-ha plots of undisturbed forest that we designated as “buffers” (see Figure 2 for plot design). We included buffers to examine potential edge effects of disturbances. Buffers were not spatially independent from disturbed treatments, so we compared them to controls in separate but identical analyses.

Disturbances were designed to emulate natural processes that spanned the range of potential forest disruptions in the Appalachian region and were implemented via timber harvest in the fall of 2006 and spring of 2007. Light treatments (least intense disturbances) mimicked stands disrupted by multiple small tree-fall gaps; we reduced basal area (BA) and overstory canopy cover (CC) on these treatments by approximately 20% (residual BA = 21±1 [SE] m^2^/ha; residual CC = 61±6 [SE] %). Intermediate treatments mimicked more severe natural disturbances such as fire, windthrow, or larger tree fall gaps; here we reduced BA and CC by approximately 40% (residual BA = 14±1 [SE] m^2^/ha; residual CC = 45±6 [SE] %). Heavy treatments emulated the most severe natural disturbances such as more intense fire and windthrow, ice-storms, or landslides; we reduced BA and CC by 75% (residual BA = 6±1 [SE] m^2^/ha; residual CC = 18±4 [SE] %). We left control plots and buffers undisturbed throughout the duration of the study (BA = 27±1 [SE] m^2^/ha; CC = 73±5 [SE] %). We attempted to apply disturbances uniformly across all treatment plots and overstory tree species composition was largely unchanged after disturbances were implemented [49]. Residual stands on the intermediate and heavy treatments were comprised of dominant and co-dominant trees. Because cerulean warblers prefer productive slopes [29], [48], plots were predominantly placed on north- or east-facing slopes to maximize warbler presence and to control for potential interactions between aspect and response.

### Territory density response

We used a before-after-control-impact study design to evaluate changes in territory density in response to treatments. We delineated and quantified territories of cerulean warblers using the spot-mapping technique. Because male warblers sing often and are easily detectable in all habitat types, spot-mapping is an ideal form of estimating density for this species. We performed eight morning censuses (from sunrise to 1030) per plot during the height of each breeding season (1 May to 15 June), 2005–2010 (two years pre-disturbance and four years post-disturbance). On gridded maps, we recorded all locations of male vocalizations including all instances of counter-singing among neighboring males, as well as any other territorial behaviors. We defined territories as geographic clusters of two or more registrations from different spot-mapping sessions and used counter-singing or other territorial behavior when available to help separate adjacent territorial individuals [50]. We also used nest and banding data (see below) to refine spot-mapping data and to validate delineation and estimation of territory numbers. We assigned fractions of territories to individuals whose territories only partially occurred within the borders of a plot (based on the proportion of registrations that fell within the plot).

We estimated baseline territory density on plots by calculating the mean density of pre-disturbance spot-mapping data (2005–06). We first compared density between the two pre-disturbance years using repeated-measures ANOVA. We performed pre-disturbance spot-mapping on MON and WYO sites in 2006 only, so these sites were not included in this pre-disturbance analysis. If we found no significant year effects, we used mean pre-disturbance density (of 2005 and 2006) as a starting point for subsequent analyses. We estimated change in territory density from pre- to post-disturbance by calculating
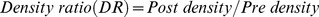
where we defined density as the number of territorial males/10 ha. Two plots were unoccupied pre-disturbance so we replaced zero values with 0.25 (the lowest recorded territory density other than zero) to estimate DR; this resulted in more conservative rates of increase than in reality, but had no effect on our inferences. Values of DR were log-transformed to meet parametric assumptions of normality and equal variance.

We analyzed this experiment as a randomized complete block design with sites treated as blocks. We compared log DR among treatments using a repeated measures mixed-model ANOVA with treatment, year, and treatment x year modeled as fixed effects and site and site x year as random effects. Year was modeled as a fixed effect because we were interested in whether treatment effects were contingent on the number of years since disturbance. If we found a main effect of treatment, we performed pairwise contrasts to evaluate differences among treatments and controls. To examine edge effects, we performed a separate, but identical, analysis to compare changes in density in buffers vs. control plots. We found no statistical difference in log DR among buffers of the three treatment types in any year (one-way ANOVA; *P*>0.30 in all years), so we used the mean density of the three buffers in this analysis.

### Age structure and body condition

To compare age structure and body condition of individuals occupying territories in each treatment type, we captured male cerulean warblers using mist-nets while broadcasting territorial songs and call notes during the height of the breeding season (May and June) during 2008–2010 (all post-disturbance). We aged males as second-year (SY; first breeding season) or after-second-year (ASY) by molt limits (particularly useful is that SY birds retain brownish juvenile primary coverts and typically two juvenile alula feathers) [51]. We measured wing chord to the nearest 0.5 mm and mass to the nearest 0.1 g. We then assigned each male to a single treatment that best reflected the individual's territory location based on evidence gathered from spot-mapping and nest searching efforts (described below). Birds were captured and aged at REMA, SQ, RB, LW, and WYO and weighed at REMA, SQ, and RB.

We compared age structure of male warblers among treatments against a null hypothesis of no difference in proportion of SY males using Pearson's chi-square tests for all sites pooled and for each region (north or south) separately (to determine if regional variation existed). To evaluate the impact of disturbance in general and to increase power, we also compared the age structure of birds captured in a disturbance of any kind (pooled) with birds captured in controls. To examine edge effects, we performed a separate, but identical, analysis to compare age structure in buffers versus controls. No difference in age structure existed among buffer types (all sites pooled: χ^2^_2_ = 0.69, *P* = 0.71; north: χ^2^_2_ = 0.01, *P* = 0.90; south: χ^2^_2_ = 1.54, *P* = 0.46), so we pooled all birds captured in buffers into a single group.

We compared body mass of males occupying territories on differing treatment plots using a two-way mixed generalized linear model (GLM). Individual birds were the sampling units in this instance and we specified age, treatment (light, intermediate, heavy, or control), and site as fixed factors and year as a random factor. We also included all two-way interactions and Julian capture date as a covariate. All two-way interactions were non-significant (all *P*>0.19), so we removed these terms and re-ran the GLM. If the treatment effect was significant, we subsequently conducted Fisher's LSD tests to determine where differences existed (at α = 0.05 and 0.10). We used body mass as an indicator of condition in this analysis because mass is often more closely related to the amount of nutritional reserves than unverified indices [52], [53]. However, to be certain this did not affect our inferences, we also calculated wing-mass residuals and found them highly correlated to body mass (*r* = 0.93); we performed analyses with both measures and found no difference. To examine edge effects, we performed a separate, but identical, analysis to compare body condition of males in buffers versus control plots. No difference in body condition existed among individuals occupying different buffer types (*F_2,20_* = 0.60, *P* = 0.56), so we pooled all birds captured in buffers into a single group. If individuals were captured in more than one season, we randomly selected one capture event to use in the analysis.

### Reproduction

We searched for nests during the entire breeding season (late April to late June), 2008–2010. We used female behavioral cues during building and incubation, and to a lesser extent male vocalizations and behavior, to locate the majority of nests. Because we were more efficient at locating nests on disturbed treatment plots, we stratified our search efforts by increasing the time spent searching on controls and buffers (in an attempt to locate an equal proportion of nests on each plot). We were unable to examine the contents of nests until nestlings were approximately 5 d old, and therefore considered nests ‘active’ when we observed parental activity at the nest that indicated egg or nestling presence (incubation or provisioning). Once active, we monitored nests every 1–3 d until fledging or confirmed nest failure occurred. From nestling day six until fledging, we monitored nests daily whenever possible for ≥30 min using spotting scopes equipped with 20–60× magnification eyepieces to count the number of nestlings present. As cerulean warbler nestlings near fledging age, they become increasingly restless (climbing over each other, begging, and preening incessantly) and are often easily counted, particularly on the steep slopes of our field sites (T.J. Boves *pers. obs.*). To conclusively determine nest fate and number of fledglings produced, we also attempted to observe fledging events. If we were unable to directly observe these events, we searched the vicinity of nests after putative fledging for parental and juvenile activity and assumed that the number of nestlings present on the last day of the nestling stage (typically day 10) to be equal to the number of fledglings produced. We considered any nest that fledged ≥1 cerulean warbler young to be successful and did not distinguish between initial and re-nesting attempts. Highly concealed nests where nestlings were difficult to count were excluded from fledgling estimates.

We initially compared logistic exposure models in Program MARK to determine the relative influence of spatial and temporal factors and treatment on daily nest survival rates (DSR). This method uses a generalized linear model with binomial distribution for each day (nest fate = 1 if failed, 0 if successful) with a logit link function to assess the influence of covariates on DSR. We compared and ranked models using a corrected version of Akaike's information criterion adjusted for small sample sizes (AIC_c_), where the minimum AIC_c_ indicates the best model (a combination of parsimony and explanatory power) [54]. We first compared models that included the spatial factors of region (southern vs. northern; RGN) and site (SITE). We found strong support for region as the spatial factor that best explained variation in DSR (when compared with region, site ΔAIC_c_ = 6.78), so we used this spatial factor alone in future models. We then compared all univariate and additive combinations of RGN, year (YEAR), and treatment (TRT), as well as YEAR×TRT and RGN×TRT interactions to test for temporal and spatial variation in treatment effects. We also included a constant survival model (NULL) for a total of 14 candidate models. We found only one nest at MON, so this site was not included in this analysis.

After this initial evaluation, we made post-hoc comparisons of nest survival rates among treatments and controls partitioned by factors determined to be influential (i.e., included in top models). We calculated cumulative survival rates for the entire nesting period by raising covariate-specific DSR to a power equal to the average length of the nest cycle (25 d) and used Program CONTRAST to determine statistical significance [55]. We approximated entire nest success variance and standard errors using the delta method following Powell [56]. We report these cumulative survival rates (hereafter, ‘nest success’) throughout the remainder of this paper for ease of interpretation. We conducted an identical analysis comparing controls and buffers to examine potential edge effects on nest success. There were no differences in reproductive success among buffers of different treatment plots in either region (north: χ^2^_2_ = 0.30, *P* = 0.89; south: χ^2^_2_ = 2.39, *P* = 0.30), so nests found in any buffer were combined into a single group.

We compared the number of fledglings produced per successful nest among treatments and controls using a mixed model ANOVA with treatment and region specified as fixed factors and year as a random factor. We again conducted an identical analysis comparing controls and buffers to examine edge effects. We used Program MARK (v6.1), JMP (v9.0), and SAS (v9.2) statistical software packages for analyses. For all statistical tests, we considered differences to be significant at *P*≤0.05 and marginally significant at 0.05<*P*≤0.10. We report means ± 1 SE.

### Source-sink modeling

We employed a deterministic population model, following Buehler et al. [47], to explore how the reproductive consequences of our treatments may affect regional source-sink dynamics. Input parameters included regionally and treatment-specific nest success and number of young produced/successful nest (as we detected regional variability in reproductive output, see results) derived from this study, as well as external estimates of after-hatch-year (AHY) and hatch-year (HY) survival, proportion of individuals that attempt to re-nest after failing, and number of re-nesting attempts. Because we were specifically interested in assessing how the reproductive consequences of disturbance may impact source-sink dynamics, we assumed equal annual survival rates, proportion of re-nesting, and number of re-nesting attempts across treatments and regions. We were unable to obtain reliable adult survival estimates from our study, likely because of high dispersal rates between breeding seasons [57], so we compared two published adult annual survival rates: 54% from Ontario [58] and 65% from Venezuela on their wintering grounds [59]. No data exist for cerulean warbler HY survival, so we assumed HY to be half of AHY survival, as has been used in previous models and has been found empirically in other passerines [60], [61]. We recognize that variation in breeding habitat may lead to differential carry-over effects on migratory or winter survival rates [62], however, we observed within-breeding season survival to be nearly 100%, and parents and offspring often dispersed from their chosen breeding habitat soon after fledging occurred (T.J. Boves, *unpub. data* and *pers. obs.*). Thus, it is likely that variation in breeding habitat had a greater impact on reproduction than on these other parameters (which were likely more highly influenced by post-breeding habitat decisions).

## Results

### Territory density

**Figure 3:**
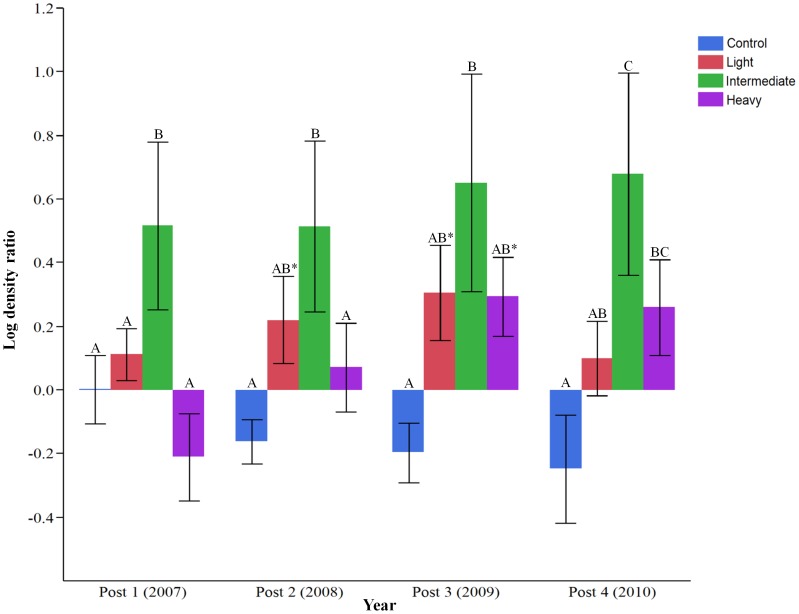
Breeding density ratio (post/pre-disturbance, log-transformed) of cerulean warblers on plots disturbed by various intensities of timber harvest. Log density ratio = 0 reflects no change in density; all values above 0 indicate increased density, all values below indicate density reduction. Different letters indicate significant differences (*P*≤0.05) among respective treatments for a given year, based on independent contrasts. Asterisks indicate marginal differences (0.05<*P*≤0.10) between respective treatment and control for a given year. Error bars represent ± 1 SE.

We found no significant year effects (*F_1,16_* = 0.05; *P* = 0.41) or year x plot interaction (*F_3,16_* = 0.16; *P* = 0.49) on pre-disturbance densities, so we used mean pre-disturbance density as a single pre-treatment value. After disturbance, we found a main treatment effect on log DR (*F_3,18_* = 4.96, *P* = 0.01) and also a treatment x year effect (*F_9,72_* = 2.79, *P* = 0.007), so we performed contrasts to evaluate differences for each year independently. In 2007 (first year post-disturbance), log DR was significantly greater on intermediate treatment plots than on all other treatment and control plots, and marginally greater on light treatment plots when contrasted with heavy (Figure 3, Table 1). In 2008, log DR remained significantly greater on intermediate treatment plots than on control and heavy treatment plots, and was marginally greater on light treatment plots than on control plots (Figure 3, Table 1). In 2009, log DR was significantly greater on intermediate treatment plots, and marginally greater on heavy and light treatment plots, when contrasted with controls, but there were no differences among any of the disturbed treatments (Figure 3, Table 1). As of 2010, log DR was significantly greater on intermediate treatment plots than on control and light treatment plots, and for the first time, was significantly greater on heavy treatment plots than on control plots (Figure 3, Table 1). Additionally, in 2010 there was no longer a statistical difference between light treatment and control plots and only a marginal difference between heavy and intermediate treatment plots (Figure 3, Table 1). We also found evidence of an edge effect as log DR was significantly greater on treatment plot buffers than on control plots (Table 1); there was no treatment x year effect in this case (*F_3,36_* = 0.88; *P* = 0.46).

**Table 1:** Density of cerulean warbler territories (± 1 SE) and results of independent contrasts comparing log density ratio (post/pre-density) of treatment plots with controls for each given year.

Treatment	Year	Density	*Df*	*F*	*P*
Control	Pre-disturbance	4.82±1.59			
	2007	4.70±1.20			
	2008	3.43±1.27			
	2009	4.16±1.84			
	2010	4.52±1.89			
Light	Pre-disturbance	5.52±1.92			
	2007	7.14±2.40	1,18	0.41	0.53
	**2008**	**7.89±2.07**	**1,18**	**3.25**	**0.09**
	**2009**	**9.11±2.70**	**1,18**	**3.96**	**0.06**
	2010	6.93±2.56	1,18	2.11	0.16
Intermediate	Pre-disturbance	4.95±2.34			
	**2007**	**7.43±2.18**	**1,18**	**8.93**	**0.008**
	**2008**	**8.07±2.06**	**1,18**	**10.16**	**0.005**
	**2009**	**11.43±3.43**	**1,18**	**11.25**	**0.003**
	**2010**	**10.57±3.02**	**1,18**	**15.03**	**0.001**
Heavy	Pre-disturbance	2.34±1.13			
	2007	1.82±1.00	1,18	1.53	0.23
	2008	3.29±1.53	1,18	1.20	0.29
	**2009**	**4.75±1.98**	**1,18**	**3.76**	**0.07**
	**2010**	**5.21±2.66**	**1,18**	**4.50**	**0.05**
Buffers	Pre-disturbance	4.81±1.33			
	**2007–2010**	**5.11±0.58**	**1,6**	**6.08**	**0.05**

Densities displayed are untransformed no. of territories/10 ha. Significant (*P*≤0.05) or marginal (0.05<*P*≤0.10) results are in bold. Buffers and controls were compared in a separate analysis with no significant treatment x year interaction, so individual annual contrasts were not performed.

### Age structure and body condition

In total, we captured and aged 204 male cerulean warblers; 27% were SY birds, 73% ASY. With all sites pooled, there was no difference in the age structure of males occupying the various treatment and control plots (χ^2^ *_3_* = 1.03, *P* = 0.79). There was also no difference in the age structure of males occupying any disturbed treatment plot vs. males occupying control plots (χ^2^ *_1_* = 0.05, *P* = 0.83). Assessing each region separately, no difference in age structure existed among treatment and control plots (north: *n* = 58, χ^2^ *_3_* = 0.64, *P* = 0.89; south: *n* = 67, χ^2^ *_3_* = 3.78, *P* = 0.29) or when all disturbed treatment plots were compared with control plots (north: χ^2^ *_1_* = 0.09, *P* = 0.93; south: χ^2^ *_1_* = 0.05, *P* = 0.82). No edge effect was observed as age structure of birds occupying buffers did not differ from those occupying control plots when all sites were pooled (χ^2^ *_1_* = 0.17, *P* = 0.68), or within regions (north: χ^2^ *_1_* = 1.18, *P* = 0.28; south: χ^2^ *_1_* = 0.36, *P* = 0.55).

**Figure 4:**
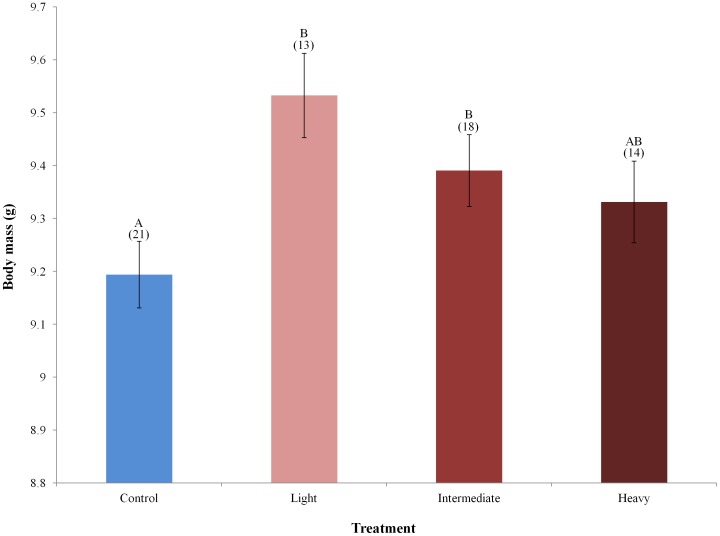
Body mass of male cerulean warblers by treatment after controlling for age, year, and site, 2008–10. Different letters indicate significant differences (*P*≤0.05) between respective treatments. Error bars represent ± 1 SE and numbers above bars indicate sample size.

Controlling for site, age, and year effects, body condition of male warblers differed by treatment (*F*_3,*56*_ = 3.41, *P* = 0.02, Figure 4). Males occupying territories on light and intermediate treatment plots were in significantly better condition than those occupying control plots (Fisher's LSD, *P*≤0.05; Figure 4) and males occupying light treatment plots were in marginally better condition than those occupying heavy treatment plots (Fisher's LSD, P≤0.10). Body condition also differed by age (SY males = 9.21±0.07, *n* = 17; ASY males = 9.52±0.04, *n* = 49; *F_1,56_* = 12.19, *P* = 0.001) but did not differ by site (*F_2,56_* = 0.82, *P* = 0.45). No edge effect was detected as body condition of males occupying buffers did not differ from those on control plots (Controls = 9.26±0.84, *n* = 21; Buffers = 9.18±0.72, *n* = 29; *F_1,42_* = 0.49, *P* = 0.49).

### Reproduction

We found and monitored 413 nests for a total of 6,384 exposure days. All four of the top models included treatment (as well as region) and the top model (RGN+YEAR+TRT) was 96× more supported than the simpler model that did not include treatment (RGN+YEAR; Table 2). There was some support for a region x treatment interaction as it was included in the third- and fourth-ranked models, but virtually no support existed for a year x treatment interaction as it was not included until the seventh-ranked model (ΔAIC_C_ = 11.97). Confidence intervals (95%) of β coefficients from the top model for the northern region (negative slope), control treatment (positive slope), light treatment (negative slope), and for 2009 (negative slope) did not include zero, which suggests their importance in explaining variation in DSR (Table 3).

**Table 2:** Model selection results for factors influencing daily survival rate of cerulean warbler nests.

Model	*k*	AIC_C_	ΔAIC_C_	*w*
S(RGN+YEAR+TRT)	7	1142.28	0	0.535
S(RGN+TRT)	5	1143.24	0.96	0.331
S(RGN+YEAR+TRT+RGN*TRT)	13	1146.23	3.95	0.074
S(RGN+TRT+RGN*TRT)	11	1147.01	4.73	0.050
S(RGN+YEAR)	4	1151.42	9.14	0.006
S(RGN)	2	1153.74	11.46	0.002
S(RGN+TRT+YEAR+TRT*YEAR)	18	1154.25	11.97	0.001
S(TRT+YEAR)	6	1156.39	14.11	0.001
S(SITE)	6	1157.43	15.15	0.000
S(RGN+TRT+YEAR+TRT*YEAR+RGN*TRT)	25	1157.80	15.52	0.000
S(TRT)	3	1159.81	17.53	0.000
S(YEAR)	3	1165.55	23.27	0.000
S(TRT+YEAR+TRT*YEAR)	16	1168.18	25.90	0.000
S(NULL)	1	1168.62	26.34	0.000

Models with a lower ΔAIC and a greater AIC_c_ weight have greater support. Model weight (*w*) and number of estimated parameters (*k*) are indicated.

**Table 3:** Parameter estimates (on logit-link scale), standard errors (SE), and 95% confidence intervals (CI) from top-ranked model (RGN+YEAR+TRT) estimating daily survival rate of cerulean warbler nests.

Parameter	β estimate	SE	Lower 95% CI	Upper 95% CI
Intercept	3.7735	0.2383	3.3064	4.2407
RGN_north_	−0.7191	0.1823	−1.0764	−0.3618
TRT_control_	0.7873	0.3372	0.1263	1.4482
TRT_light_	−0.5395	0.1949	−0.9216	−0.1574
TRT_intermediate_	0.3610	0.2682	−0.1646	0.8866
YEAR_2008_	−0.2521	0.2260	−0.6950	0.1908
YEAR_2009_	−0.4299	0.1989	−0.8197	−0.0400

**Figure 5:**
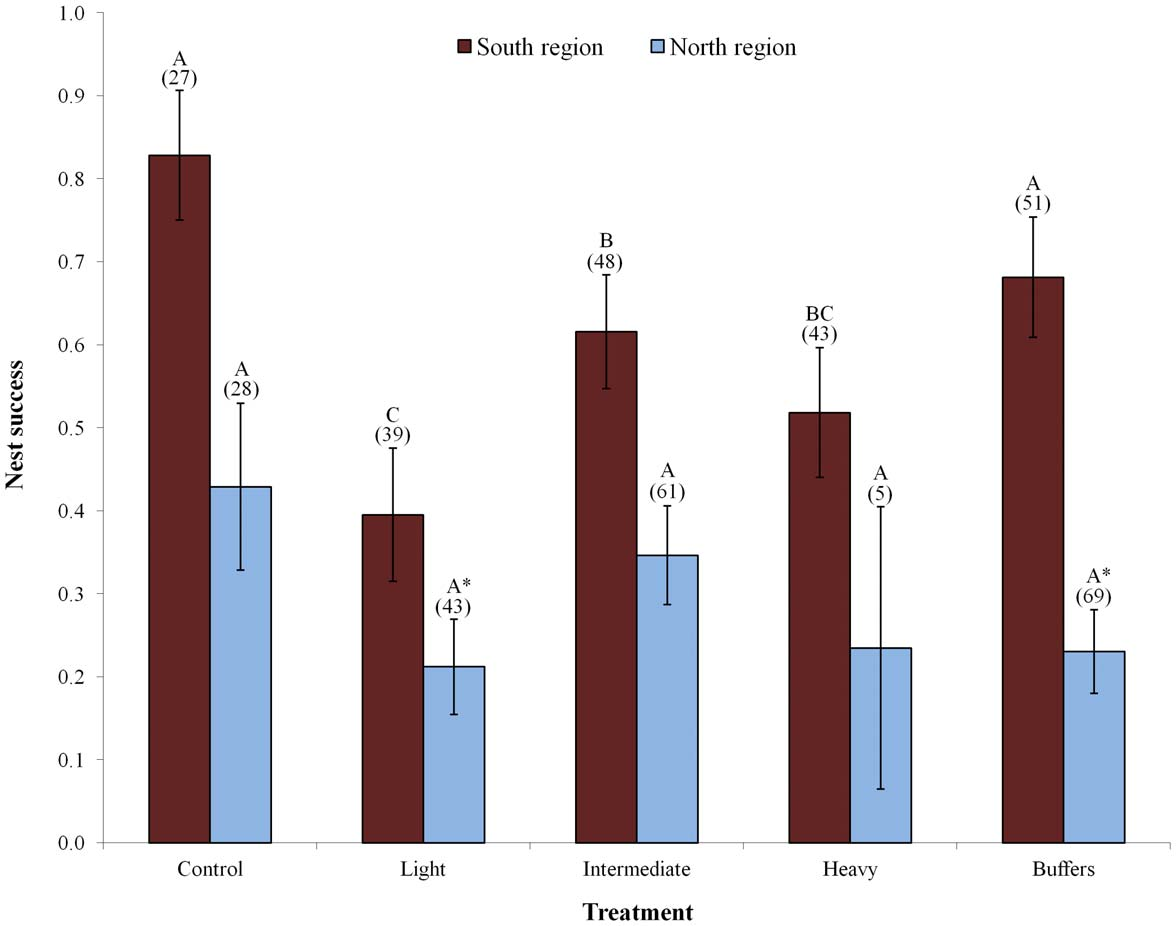
Cerulean warbler nest success by treatment and region, 2008–10. Different letters indicate significant differences (*P*≤0.05) between respective treatments within a region (based on CONTRAST χ^2^ test). Asterisks indicate marginal differences (0.05<*P*≤0.10) between respective treatments and controls within a region. Markings above buffer columns refer only to their relationship with controls. Error bars represent ± 1 SE and numbers above bars indicate nest sample sizes.

Cumulative nest success differed among all sites (χ^2^_5_ = 27.56, *P*<0.0001) but did not differ among sites within regions (North: χ^2^_3_ = 1.61, *P* = 0.66; South χ^2^_1_ = 1.44, *P* = 0.23). Thus, we pooled nests from respective regions to further assess treatment effects on nest success. In the southern region, cumulative annual nest success varied from 0.48±0.06 in 2009 to 0.67±0.05 in 2010. When pooling nests from all three years (Figure 5), nest success in this region was greater on control plots than on light (χ^2^_1_ = 15.02, *P*<0.0001), intermediate (χ^2^_1_ = 4.41, *P* = 0.04), and heavy treatment plots (χ^2^_1_ = 15.02, *P*<0.0001). Nests on intermediate treatment plots were more successful than those on light treatment plots (χ^2^_1_ = 4.38, *P* = 0.04). There was no evidence of an edge effect on nest success as controls and buffers did not differ (χ^2^_1_ = 1.89, *P* = 0.17). Annually, nest success was greater on control plots than heavy treatment plots in 2009 (χ^2^_1_ = 26.07, *P*<0.0001) and greater than light treatment plots during 2009 (χ^2^_1_ = 33.73, *P*<0.0001) and 2010 (χ^2^_1_ = 5.64, *P* = 0.02).

In the northern region, annual nest success ranged from 0.22±0.04 (2009) to 0.40±0.06 (2010). When pooling nests from all three years (Figure 5), nest success was marginally greater on control plots than on light treatment plots (χ^2^_1_ = 3.50, *P* = 0.06), but did not differ among any other pairwise combination of treatments and controls. There was marginal evidence of an edge effect as nests on control plots were marginally more successful than those on buffer plots (χ^2^_1_ = 3.12, *P* = 0.08). On an annual basis, nest success did not differ between control or any treatment or buffers (all *P*>0.10), however small sample sizes hampered our ability to detect statistical differences annually.

**Figure 6:**
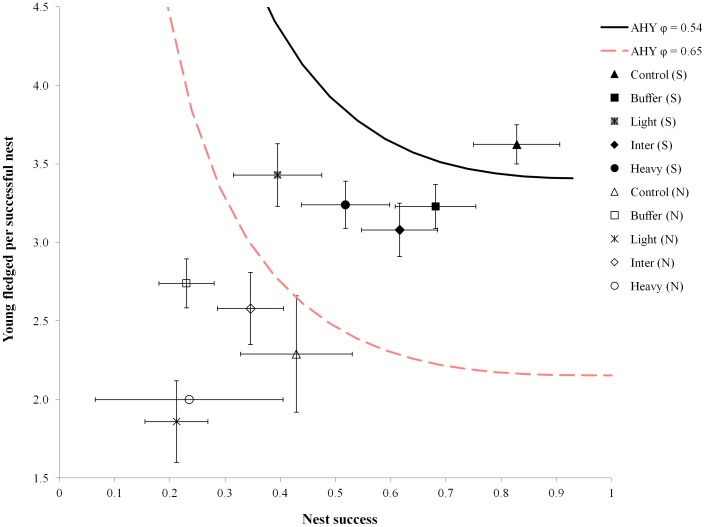
Graphical model of cerulean warbler source-sink dynamics in relation to regional reproductive consequences of emulated disturbances. We used point estimates of nest success and mean number of young fledged/successful nest on various treatments from the southern (S) and northern (N) regions, 2008–10. Error bars indicate ±1 SE. Two possible lambda threshold curves are displayed, each based on a published annual survival rate for cerulean warblers: (1) from Ontario (54% AHY survival), and (2) from Venezuela (65% AHY survival). Points to the left of (or below) the threshold curve, for each given survival rate, represent decreasing, or sink populations, and points to the right of (or above) the curve represent increasing, or source populations. HY survival was considered to be 0.5 of the AHY rate and three re-nesting attempts were assumed to occur for all failed nests.

The number of fledglings produced/successful nest differed by region; warblers in the south produced more fledglings/successful nest (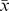 = 3.33±0.07) than in the north (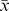 = 2.28±0.14; *F_1,97_* = 33.98, *P*<0.0001; see Figure 6). However, there was no effect of treatment (*F_3,42.95_* = 0.64, *P* = 0.60). Comparing controls with buffers, nests in the south again produced more young (*F_1,73.85_* = 19.04, *P*<0.0001), but there was no evidence of an edge effect on fledglings produced (*F_1, 73.05_* = 0.05, *P* = 0.82).

The cause of nest failure was directly observed or inferred from evidence at only 36 (of 174 failed) nests. Predation was the main cause of nest failure (*n* = 22 of these 36 nests) followed by disease or starvation (*n* = 6). The majority of failed nests were abandoned suddenly for unknown reasons, suggesting that predation was most likely, but nest desertion subsequent to brown-headed cowbird (*Molothrus ater*) parasitism cannot be ruled out.

### Source-sink dynamics

Our graphical model shows that given an AHY annual survival rate of 54%, only control plots in the southern region had levels of reproduction sufficient to maintain a stable (or source) population (Figure 6). If annual survival was increased to 65%, all treatment plots in the southern region would act as sources (λ>1). We found no treatment plot in the northern region, including controls, that could maintain a stable population given either of these two survival rates; all require either greater annual survival or reproductive output, immigration from other locations, or an adjustment in model assumptions to persist.

## Discussion

We hypothesized that existing second-growth forest in the eastern United States may not provide quality habitat for some late-successional species, especially if those species are adapted to small-scale natural disturbances that have been altered or suppressed within contemporary forests. Accordingly, we documented attraction to emulated disturbances of various intensities by a declining species typically considered to be late-successional, the cerulean warbler, in highly-forested ecosystems in the Appalachian Mountains. The density response we observed is congruent with recent correlative studies that found cerulean warblers associated with canopy disturbances within mature forests [21], [29]. In our study, attraction was greatest after intermediate and heavy disturbances, suggesting that the species is adapted to fire, intense windthrow, landslides, or other moderate interior natural disturbances, rather than smaller single tree-fall gaps caused by tree senescence, for instance. Density increases after intermediate disturbances on some sites were unexpectedly strong and immediate (e.g., 0.25 territories pre-disturbance to 8.5 territories in the first breeding season post-disturbance at LW); on other sites increases were more modest, perhaps because of pre-disturbance saturation. At RB, pre-disturbance density was at a (likely) near-saturation level of 17 territories/10 ha. Density increased on this plot post-disturbance, but only to a maximum of 20.5 territories in 2010. At such great pre-disturbance densities, it would seem unlikely that many more birds could occupy the area, no matter how attractive the habitat became. Densities increased more gradually after heavy disturbances (and actually decreased in the first year post-disturbance). This suggests that some physiognomic cue important for habitat selection required multiple growing seasons to develop after these more severe disturbances, and could be related to temporal changes in canopy or understory structure [63]. The edge effect that we detected (i.e., density increases in undisturbed buffers surrounding disturbed plots) was primarily related to an increase in birds establishing territories that overlapped both the treatment plot and buffers (J. Sheehan, *unpub. data*).

As disturbances attracted warblers at higher densities, the lack of difference in age structure among treatments runs counter to the expectation that older birds should out-compete inexperienced males and settle in preferred habitat more often [64], [65]. However, we did find that males occupying light and intermediate treatment plots, regardless of age, were in better condition than those inhabiting controls. We do not know if this difference reflects a settlement bias (e.g., if individuals on disturbed treatments were in better condition on arrival or of higher quality), if disturbances allowed individuals to improve their condition (e.g., by virtue of increased insect availability after disturbances), or if a combination of the two was responsible for this pattern. Canopy gaps can alter the composition of arthropod communities [66] and cerulean warblers may be better adapted for foraging on invertebrate species inhabiting broken canopies. Indeed, George [49] found that warblers increased their use of aerial foraging maneuvers after partial timber harvests occurred. However, it is not known if this behavioral alteration results in improved condition; future studies that monitor settlement patterns and individual changes in body mass across habitat types would help tease these possibilities apart.

Despite the density increases and improved body condition of individuals occupying treatment plots, per capita reproductive output was lower on many of the treatment plots compared to local control plots. Reproductive differences were most obvious in the southern region, where disparities in nest success between control and treatment plots were statistically apparent in all cases. In the northern region, factors seemingly unrelated to the manipulations reduced overall reproductive success to where disturbance had less influence, and low sample sizes made it difficult to detect statistical differences in some instances (e.g., *n* = 5 nests on heavy treatment plots). However, nest success was marginally greater on control plots than on light treatment plots (and buffers) in this region as well. Thus it appears that individuals, particularly in the southern region, often chose to breed in habitats where they failed to maximize reproduction.

There are numerous potential explanations to this seeming contradiction [see 67 for an exhaustive list]. One possibility is that by breeding in disturbed habitats, individuals increased their lifetime fitness (despite reductions to current reproductive output) by improving their chances of surviving to the next breeding season or by improving their offspring's chances of survival during the dangerous post-fledging period. Increased annual survival of cerulean warblers after canopy disturbances may be possible by virtue of the potential carry-over effects of improved body condition on migratory or winter survival [62], [68], [69], and post-fledging survival rates may be greater because of the abundance of concealing understory vegetation on intermediate and heavy treatment plots [70], [71]. However, as alluded to previously, the influence of breeding habitat on these future components of fitness may be relatively indirect and is currently unclear, while the influence of breeding habitat on nest success and fledgling production is direct and obvious. A second possibility is that density was not an accurate reflection of habitat preference and individuals were forced into disturbed habitats via competitive exclusion by more dominant individuals [39], [65]. The evidence does not support this possibility however, as we documented no age differences among individuals occupying treatment and control plots, and those individuals that did occupy territories in disturbed habitats were, in fact, in better condition than those in undisturbed control plots.

A third possibility is that individuals may have made maladaptive decisions when selecting disturbed habitats (i.e., disturbed interior forest stands may act as “ecological traps” [72]), particularly when choosing among habitats at the local scale. Under evolutionarily-relevant historical conditions, canopy disturbances in old-growth forests caused by fire or natural treefalls may have created habitats where warblers were able to achieve relatively high levels of fitness. After emulated natural disturbances, environmental cues associated with high fitness may still elicit the same habitat selection behavior, however other conditions, contemporary in nature, may have also been altered, thereby potentially decoupling the habitat cues from historically high levels of reproduction. If broad-scale factors (such as landscape-scale fragmentation) [73] are responsible for altering the ecological pressures that are at play, then the source of disturbance may be unimportant as even natural disturbances may result in maladaptive behavior. In response to a natural disturbance event, Jones et al. [74] reported a decrease in cerulean warbler nest success a year after an ice storm in Ontario, Canada. However densities also declined in that case, likely producing a sink rather than a trap. Thus, despite our best intentions, forests disturbed by human activity may only resemble naturally disturbed forests, but may differ in terms of tree age-class distribution [75], increased soil disturbance [76], a lack of standing dead trees or snags [77], or in spatial scale and canopy structural complexity [6]. These artificial modifications may result in differing predation pressures, arthropod composition [78], or other factors that may make it difficult for warblers to correctly assess habitat quality. Potential ecological traps created by timber harvests have recently been identified for other declining species including olive-sided flycatchers (*Contopus cooperi*) breeding in selectively logged forests in Montana [43] and rusty blackbirds (*Euphagus carolinus*) breeding in regenerating clear-cuts in northern New England [79]. In the future, research evaluating survival during the post-fledging period across disturbance gradients is warranted for cerulean warblers (and other canopy nesting species), although this work will be challenging because of difficulties in capturing nestlings and fledglings. In addition, comparisons of selective pressures in natural versus emulated disturbances and 24-hour video surveillance of nests, will improve our understanding of the causes of nest failure and adaptive nature of habitat selection behavior.

An important caveat of our study is that we measured responses that were short-term in nature (1–4 years), and responses may vary over time. We may have even observed an adjustment in habitat selection behavior in 2010, only four years post-disturbance. While densities increased in 2008 and 2009 on the light treatment plots, by 2010 the density response to light treatments was no longer statistically different than the response to controls. Birds may track variation in breeding success and adjust their habitat selection decisions to match local conditions [80], [81]. If habitat selection behavior is dynamic, and relatively low levels of nest success persist on disturbed treatments, densities on light (and possibly other) treatment plots may eventually drop below densities on the control plots, but this hypothesis will require further study. An alternative explanation is that some canopy closure had already occurred on the light treatment plots [e.g., 82], and attraction to the resulting structural features of the vegetation had begun to wane. Continued monitoring of these field sites to assess the persistence of the trends we have observed would be very useful.

### Conservation and Management Implications

The conservation and management implications of our results are complicated by the spatial variability of the impact of disturbances on reproduction, and regional variation in reproductive output in general. In previous studies that have documented putative maladaptive habitat selection, preference has only been considered at local scales (e.g., between adjacent habitats; [43], [83]–[85]). However, for migratory or highly dispersive species, habitat selection behavior also occurs at broader scales (e.g., the decision to breed in the northern or southern portion of the range) [86]. Thus, simply comparing choices made during the final stages of habitat selection greatly simplifies, and possibly misrepresents, this complex behavioral process. In the case of cerulean warblers, although our results suggest that preference for disturbed forest may be maladaptive at the local scale in the southern region, selection for disturbed habitat in this region could actually be adaptive if the alternative option was to migrate further north to breed, or to not reproduce at all. Therefore, a fundamental question that affects our interpretations, as well as those of any study of habitat selection that assesses the adaptive nature of this behavior, is: what alternative breeding locations do birds forego to breed in attractive habitat types? As cerulean warblers appear to regularly engage in long-distance dispersal (putatively searching for recently disturbed forest habitat), the creation of attractive habitats in the southern region (the Cumberland Mountains) may actually be beneficial to the overall sustainability of the global population because it could provide additional breeding opportunities in this highly productive region. However, for this management strategy to be successful, it requires that birds attracted to disturbances in the Cumberlands to have otherwise attempted to breed in less productive regions (e.g., the northern region), or not at all (i.e., ‘floaters’), rather than breeding in local undisturbed forest.

In the northern region, emulating disturbances did not always result in major declines in local reproductive success and thus doing so may not create traditional ecological traps. However, if newly created disturbances in this region attract birds from distant locations where fecundity may have been greater (e.g., Cumberlands), a broader-scale trap could be created. Again, if individuals attracted to disturbances in the north would have otherwise failed to reproduce at all, even these northern disturbances with relatively low per capita productivity could have a positive population effect. These contingencies demonstrate how the true impact of putative ecological traps may be quite complex and difficult to assess when viewed in isolation.

Despite those complexities, our study provides evidence that increasing, or even maintaining, populations of cerulean warblers, and potentially other disturbance-adapted late-successional species, into the future will likely require a cooperative, landscape-scale approach to managing forests. The challenge for conservation will be to determine the appropriate locations for implementing disturbances on the landscape in order to provide habitat for a maximum number of breeding pairs while maintaining maximum individual productivity. Accordingly, a conservative approach to management is warranted which would involve emulating disturbances similar in scale and intensity to our intermediate treatments in locations where existing forest structure is unsuitable and breeding densities are low, while limiting disturbance in areas where forest structure is currently appropriate and breeding densities are higher. Determining where appropriate forest structure currently exists may be accomplished by performing systematic bird surveys (to directly assess density) or by applying predictive models which use vegetative and topographic measurements [similar to 21], [48]. Future studies examining annual survivorship and long-distance dispersal patterns of cerulean warblers inhabiting various disturbed treatments in multiple regions could help inform this situation further. Finally, it is important to note that we found only minimal impacts of disturbance, beneficial or otherwise, extending beyond the borders of the area treated (i.e., buffers), which suggests that the consequences of any of the forest management practices evaluated here will mostly apply only to the harvested stands themselves.
